# Social mobility and class segregation in personal networks over time: evidence from longitudinal data

**DOI:** 10.3389/fsoc.2026.1782127

**Published:** 2026-08-03

**Authors:** Gabriel Otero, Alejandro Plaza, Ignacio Zavala-Aránguiz

**Affiliations:** 1 School of Sociology, Universidad Diego Portales, Santiago, Chile; 2 RTG DYNAMICS, Humboldt-Universitat zu Berlin, Berlin, Germany; 3 Instituto de Sociología, Pontificia Universidad Catolica de Chile, Santiago, Chile

**Keywords:** Chile, homophily, intragenerational mobility, position generator, social class

## Abstract

This study examines how class-based network homogeneity evolves over time and whether intragenerational social mobility reshapes personal network composition in Chile. Drawing on four waves of longitudinal data from the Chilean Longitudinal Social Survey (ELSOC, 2016–2023), we assess the extent to which class stability and different forms of social mobility—conjunctural, structural, and cumulative—influence the degree of class homogeneity in personal networks. We use the position generator instrument to measure cross-class social ties across 13 occupations, calculating the proportion of ties belonging to respondents’ own class. Our findings reveal that class homogeneity remained relatively stable across the period (40–45%), with both upper-middle-class and working-class respondents reporting similarly high proportions of same-class contacts (above 40%). Class-stable individuals consistently exhibit higher network homogeneity than mobile individuals, supporting theoretical expectations regarding cumulative homophily effects. Upwardly mobile individuals generally display lower homogeneity than downwardly mobile individuals in short- and medium-term transitions, although this asymmetry becomes less consistent when cumulative mobility trajectories are considered. Stable lower-middle-class individuals exhibit significantly lower homogeneity than stable working-class or upper-middle-class respondents. Long-term mobility patterns—whether upward, downward, or oscillatory—correspond to more diverse social networks compared to stable class locations, with less consistent differences between mobility directions. These results demonstrate that class boundaries, while permeable through mobility, continue to have a bearing on the structure of social relationships, with class-based network segregation reflecting deeply embedded structural and cultural mechanisms that resist transformation through individual mobility alone.

## Introduction

Personal network analysis has long recognized a fundamental principle governing social connection: the tendency for similar individuals to form ties with one another, a pattern known as homophily (Lazarsfeld and Merton, 1954; McPherson et al., 2001). Far from being incidental, this principle operates as a powerful organizing force that systematically structures diverse forms of social relationships, from marriage and friendship to professional, ultimately generating network segregation within personal networks (e.g., DiPrete et al., 2011). While homophily patterns based on age, gender, education, and religion have been extensively documented and theorized (e.g., Bargsted et al., 2020; Hofstra et al., 2017; Smith et al., 2014; Völker, 2022), the dynamics of class-based segregation in personal networks remain comparatively underexplored in empirical research. This gap is particularly striking given the centrality of class in social stratification research (Bourdieu, 1984; Wright, 1997), the well-established understanding that class structures shape access to resources, opportunities, and social capital (Blau, 1977; Lin, 2001), and evidence showing that class-based segregation undermines social cohesion (Otero et al., 2022a).

The limited body of research examining class composition in personal networks has consistently revealed that individuals exhibit above-average levels of intra-class connectivity, with this tendency being particularly pronounced at the extremes of the class structure (e.g., Alecu et al., 2022; Bian et al., 2005; Wright and Cho, 1992; Otero et al., 2021). For instance, research on Chilean adults in 2016 documented mean class homogeneity levels of 44% in personal networks, with notably elevated rates among both the poorest (50%) and wealthiest (49%) classes (Otero et al., 2021). Similar patterns have emerged in studies of Chinese urban residents (Bian et al., 2005) and cross-national comparisons of class network inequality (Iturra-Sanhueza, 2026).

Despite these insights, the most critical gap in our understanding of class homogeneity within personal networks concerns its temporal dynamics. While some studies have provided evidence of changes in class heterogeneity over time (e.g., Plaza et al., 2026), virtually no research has examined how class-based network homogeneity evolves either longitudinally or across the life course. This limitation largely stems from the scarcity of longitudinal datasets that simultaneously capture socioeconomic positioning and detailed network configurations. Moreover, the few panel studies that do include network data typically lack the fine-grained occupational or income measures required for meaningful class analysis, whereas comprehensive class-based surveys rarely incorporate repeated network-position measures or position generators. This is a relevant empirical gap, as theoretical perspectives offer competing predictions regarding whether class homogeneity intensifies, stabilizes, or diminishes across biographical transitions (Blau and Schwartz, 1984; Emirbayer and Goodwin, 1994).

Existing research on personal network dynamics has largely focused on discrete life transitions and biographical events such as employment changes, marriage, divorce, parenthood, and widowhood as key catalysts of network reconfiguration (e.g., Kalmijn, 2012; Marin and Hampton, 2019; Milardo, 1987; Rözer et al., 2020). These studies demonstrate that major life course transitions reshape network composition by altering opportunity structures, time availability, and normative expectations surrounding social interaction (Feld, 1981; Mollenhorst et al., 2014). However, when linking network analysis with class analysis, patterns of social mobility, particularly individuals’ career trajectories and within-lifetime class transitions, emerge as a natural and underexplored source of network change. Classical sociological theory emphasized that social mobility poses dilemmas for interpersonal relations rather than guaranteeing social integration. Blau (1956) argued that both upwardly and downwardly mobile individuals often occupy relationally ambiguous positions, as they tend to be weakly integrated into the networks of both their class of origin and their class of destination. Mobility across the class structure exposes individuals to new occupational contexts, residential environments, and associational milieus, potentially reshaping available contact pools while weakening ties rooted in earlier class positions (Blau, 1977). Moreover, class transitions may generate cultural misalignment with prior networks, as mobility frequently involves shifts in lifestyles, values, and consumption patterns that strain cross-class relationships (Friedman, 2016; Lee and Kramer, 2013; Mallman, 2017). As a result, mobile individuals may selectively cultivate ties aligned with their destination class while distancing themselves from prior contacts, reflecting processes of instrumental networking and symbolic boundary work (Méndez and Gayo, 2019; Naudet, 2018).

Based on this background, the present study examines the extent to which class-based network homogeneity has changed or remained stable over time, and whether such patterns are influenced by processes of intragenerational mobility. Analytically, examining the relationship between social mobility and network composition may offer a productive synthesis of two central preoccupations of class analysis (Wright, 1997; Goldthorpe, 2010): mapping the structural positions individuals occupy within stratification systems and understanding the conditions under which movements between these positions occur. By investigating whether and how class trajectories reshape personal networks, we can illuminate the relational dimensions of mobility, which are frequently obscured when mobility is conceptualized purely as individual positional change (Wright and Cho, 1992).

We focus on the context of Chile, a particularly revealing case for examining class-based network dynamics. The country has one of the highest levels of income inequality in the OECD, with a Gini coefficient persistently above 0.43 (OECD, 2025), accompanied by pronounced occupational stratification and limited intergenerational mobility (Espinoza and Núñez, 2014; Torche, 2005). This structural inequality has produced sharply segregated personal networks along class lines (Garreton et al., 2023; Otero et al., 2021). Such network segregation both reflects and reinforces mechanisms of social reproduction, concentrating opportunities and resources within class boundaries (Contreras et al., 2019). Chile’s combination of high inequality, documented network segregation, and substantial within-cohort occupational mobility makes it an ideal setting for investigating how class trajectories reshape personal network composition over time.

Our research questions are as follows:

(1) To what extent does class segregation in personal networks change over time?
(2) To what extent are changes in class segregation in personal networks influenced by patterns of intragenerational social mobility?

For the analysis, we use survey data from the Chilean Longitudinal Social Study (ELSOC), designed by the Centre for Social Conflict and Cohesion Studies (COES), which represents Chilean adults living in urban areas. Specifically, we analyze data from waves 1, 3, 5, and 7 (collected between 2016 and 2023), comprising approximately 3,735 observations. To measure network segregation, we use the “position generator” instrument included in ELSOC, which asks respondents how many people they know in 13 different occupations of varying occupational status, such as lawyers, taxi drivers, doctors, and accountants. The paper continues by outlining the arguments that guide our hypotheses, followed by a section describing the data, variables, and methods. We then present the results and conclude with a discussion of the findings.

## Theoretical background

### Structural analysis of social class

Social classes are relational and hierarchical groupings within society that structure systematic inequalities in life chances and opportunities. These divisions arise from individuals’ relationships to the means of production, as well as from their occupation, educational credentials, and the degree of autonomy or supervision exercised in the workplace (e.g., Goldthorpe, 2010; Wright, 1997). A substantial body of research has shown that social class shapes a wide range of outcomes, from health and income to societal attachment and social recognition (e.g., Lareau and Conley, 2008; Otero et al., 2022a). Rather than functioning merely as statistical categories, social classes represent positions embedded within structural relations of advantage and disadvantage that, to a significant extent, persist across generations (Erikson and Goldthorpe, 1992).

The structural analysis of social class can be divided into two distinct yet interconnected tasks. On one hand, it involves identifying positions within class relations (Goldthorpe, 2010; Oesch, 2006; Wright, 1997). This dimension focuses on the relational aspects of class structure, examining how different positions generate conflicting or aligned interests, exploitation dynamics, and concentration of resources. Scholars can delineate class boundaries by analyzing employment relations, skill assets, property ownership, and organizational authority (Wright, 2015). On the other hand, class analysts examine the permeability of the boundaries that separate these positions—an aspect typically assessed through the study of social mobility patterns (Breen, 2004; Erikson and Goldthorpe, 1992). Specifically, this second task involves examining whether class positions function as rigid barriers or as fluid categories by measuring both intergenerational and intragenerational movement across class boundaries (see also Kalleberg and Mouw, 2018; Sørensen, 1975; Torche, 2015). High permeability likely suggests an open, meritocratic system, while limited mobility may indicate structural rigidity and class reproduction (Blau and Duncan, 1967).

One way to link class boundary permeability with structural class analysis is by examining class-based patterns of social interaction (Wright and Cho, 1992). Social networks serve as crucial mechanisms through which class positions are maintained or transformed, as personal contacts facilitate access to socioeconomic resources, particularly information and influence, that can significantly enhance job opportunities and status attainment (Granovetter, 1973; Lin, 1999; Mouw, 2003). The relationship between class structure and network composition can also be bidirectional. Rigid class structures may foster the development of segregated class networks, whereas greater permeability in class positions is likely to promote cross-class ties (Blau, 1977; Blau and Schwartz, 1984). This dynamic interdependence between class structure and network formation raises fundamental questions about how class-based personal networks emerge, evolve, and are sustained over time.

### The sources of class homogeneity in personal networks

There is a well-known tendency for individuals to interact more frequently with same- or similar-class others than persons outside their stratum (Otero et al., 2021). Such a tendency also applies for other categories such as gender, ethnicity, and age, and is usually known as the “principle of homophily” (McPherson et al., 2001).

Theoretical frameworks converge on two fundamental mechanisms to explain patterns of homophily in class-based social relationships (Espinoza and Otero, 2024). The first mechanism centers on structural constraints and opportunity structures that systematically channel social interaction within class boundaries. Closure-generating devices, including residential and educational segregation, exclusive organizational memberships, and gatekeeping practices, allow dominant classes in particular to regulate access to valued social settings and interaction contexts (Bourdieu and Passeron, 1977; Khan, 2011; Lareau, 2011). These structural arrangements create profoundly unequal meeting opportunity structures wherein individuals occupying similar class positions encounter one another with far greater frequency than they encounter those from different class backgrounds (Blau and Schwartz, 1984; Feld, 1981). Geographic concentration of classes in distinct neighborhoods, occupational segregation across workplaces and industries, and the stratification of leisure spaces and voluntary associations all contribute to this structural homophily, making cross-class contact statistically improbable even in the absence of any intentional preference for same-class interaction (e.g., Garreton et al., 2023; Louf et al., 2025; Otero et al., 2022b).

The second mechanism operates through cultural alignment and symbolic boundaries that shape preferences for same-class relationships. Drawing on Bourdieu (1984) seminal analysis, this perspective emphasizes that individuals occupying similar positions within the class structure develop homologous cultural repertoires, such as shared tastes, aesthetic sensibilities, linguistic styles, embodied dispositions, and lifestyle practices that constitute the habitus (Bourdieu, 1990; Lamont and Fournier, 1992). These cultural commonalities facilitate mutual understanding, ease of interaction, and affective affinity among class peers, while cultural distance across class boundaries can generate discomfort, miscommunication, and social awkwardness that discourage cross-class relationship formation and maintenance (Otero et al., 2021). Moreover, individuals actively employ cultural markers to construct symbolic boundaries that distinguish “people like us” from others, using shared cultural knowledge as a basis for inclusion and exclusion in social networks (Jarness, 2017). These boundary-making processes are not merely passive reflections of cultural difference but constitute active strategies through which groups maintain their social position and reproduce inequality across generations.

The two mechanisms, structural constraints on meeting opportunities and cultural preferences shaped by class position, are said to work in tandem to produce and sustain class homophily in social interactions. Thus, they generate strong expectations that cross-class social interactions will be limited in frequency and depth, and that individuals will consequently form personal networks characterized by substantial class segregation (Alecu et al., 2022; Bian et al., 2005; Iturra-Sanhueza, 2026; Lindh and Andersson, 2025; Otero et al., 2021).

Importantly, these homophily-generating mechanisms may operate with varying intensity across class positions, producing systematically different network configurations through fundamentally distinct processes (Otero et al., 2021). Upper classes likely exhibit the most pronounced and stable homogeneity due to their superior capacity for social reproduction through exclusive access to institutions such as elite private schools, prestigious universities, members-only social clubs, and selective business networks that explicitly screen for class markers and cultural alignment (Bourdieu and Passeron, 1977; Khan, 2011). These institutional environments engineer homogeneity by design, while simultaneously providing elites with both greater resources for boundary maintenance and stronger incentives to preserve exclusive access to valuable social capital. Middle classes may display more complex and dynamic patterns: they might experience heightened network heterophily as they traverse class boundaries and encounter diverse contact pools across educational and occupational transitions (Friedman, 2016; Jarness, 2017).

Lower and working classes may exhibit homogeneity levels rivalling those of upper classes, yet arising from profoundly different mechanisms, specifically spatial and institutional exclusion rather than deliberate boundary maintenance (Espinoza and Otero, 2024; Massey, 1996). Concentrated neighborhood poverty, residential segregation, underfunded schools, and limited labor market access can trap disadvantaged populations in homogeneous social environments with minimal opportunities for cross-class contact (e.g., Espinoza, 1999). Unlike upper-class homophily, which reflects strategic selection and resource hoarding, lower-class network homogeneity likely stems from structural constraints that limit geographic mobility, educational opportunities, and occupational diversity (Wacquant, 2008). In other words, class homogeneity in personal networks is imposed by exclusionary processes rather than chosen according to preferences (Bourdieu, 1999).

### Class mobility and changes in personal networks

In this work, we argue that patterns of intragenerational mobility can reconfigure class-based network homogeneity, particularly because it disrupts traditional mechanisms of class homogeneity formation. Indeed, intergenerational and intragenerational social mobility can be understood as life events that potentially alter the preferences and contact opportunities occurring within social interaction contexts (McPherson et al., 2001). First, upward or downward movements through the class structure necessarily expose individuals to new occupational contexts, residential environments, and associational milieus, thereby altering the composition of available contact pools while potentially weakening ties rooted in earlier class positions (Blau, 1977; Feld, 1981). Second, as individuals experience class transitions over the course of their own lives, they may encounter cultural misalignment with networks formed in previous class locations, as mobility often entails shifts in lifestyle, consumption patterns, and normative orientations that can strain relationships spanning class boundaries (Friedman, 2016; Bourdieu, 1984; Lee and Kramer, 2013; Jarness, 2017). Consequently, social mobility (upward or downward) may lead mobile individuals to manage their networks actively and strategically, prioritizing certain ties over others according to their instrumental or symbolic utility (Lin, 2001).

Building on this argument, we consider that intragenerational mobility may produce somewhat distinct network dynamics depending on the direction of movement, upward or downward. Upward intragenerational mobility opens pathways to previously inaccessible social circles and privileged networks in higher education or higher-status occupational settings, while often allowing individuals to preserve connections to their former class position (Blau and Duncan, 1967; Wegener, 1991). The result may be a temporarily dual network structure that spans class boundaries, a form of structural bridging that would be unlikely to occur through class background alone. In this sense, upwardly mobile individuals likely become brokers positioned between distinct social worlds, capable of accessing resources and information from multiple class contexts (Lin, 2001; Burt, 2005). This brokerage position, while potentially advantageous for network diversity, may introduce tensions as individuals navigate the competing expectations, norms, and social codes of different class-based networks.

However, upward intragenerational mobility may involve fundamental transformations in habitus, lifestyle, and cultural orientation that render origin-class relationships increasingly strained or obsolete (Naudet, 2018; Friedman, 2016). As upwardly mobile individuals adopt the consumption patterns, leisure preferences, and interaction styles of their destination class, they may experience growing cultural distance from origin-class contacts, leading to relationship attenuation through reduced interaction frequency and diminished mutual understanding (Jarness, 2017; Lee and Kramer, 2013). Additionally, upwardly mobile individuals may experience aspirational belonging, motivating tie formation with the destination class as a mechanism of symbolic integration and validation of their new social position (Bourdieu, 1984). This motivation often manifests in practices of cultural distinction and self-presentation oriented toward the signaling of belonging to the new group (Friedman, 2016).

At the same time, destination-class institutions such as workplaces, neighborhoods, and voluntary associations often provide dense opportunity structures for forming same-class ties while limiting cross-class contact (Khan, 2011; Lareau, 2011). Beyond cultural alignment, upwardly mobile individuals may also consolidate their achievements by building social capital appropriate to their new position, cultivating relationships that provide resources, information, and opportunities for maintaining and advancing their trajectory (e.g., Pena-López et al., 2024). Consequently, those experiencing upward career mobility may strategically cultivate ties consistent with their current or aspired class position while allowing earlier connections to attenuate, a process reflecting instrumental networking strategies and symbolic boundary maintenance pressures (Friedman, 2016; Flemmen et al., 2018). In this sense, upward mobility appears less likely to generate meaningful network diversification than to facilitate a progressive consolidation of ties oriented toward the destination class.

For those experiencing downward intragenerational mobility, class-based network homogeneity dynamics likely operate through distinct mechanisms, though potentially leading to outcomes comparable to upward mobility in terms of reduced class homogeneity. On the one hand, downward mobility may precipitate the rupture of prior relationships due to shame or social stigma. Downwardly mobile individuals may experience abandonment or distancing by origin-class contacts, particularly in contexts where status is central to group identity (Newman, 1988). On the other hand, downwardly mobile individuals may no longer possess the economic and cultural resources required to sustain participation in sociability spaces and cultural consumption associated with their origin class, generating lifestyle incompatibilities that strain existing relationships (Bourdieu, 1984). Nevertheless, individuals experiencing downward mobility may retain “residual” ties with their origin class, especially family bonds, childhood friendships, or relationships formed in educational institutions, which may persist through inertia or affective commitment despite divergent trajectories (Newman, 1988; Friedman, 2016). Such ties may function as bridging connections across class boundaries.

Regarding the formation of new ties, transitions into lower-status occupational contexts and potential residential moves to less privileged neighborhoods may involuntarily expose downwardly mobile individuals to new interaction settings in which contact opportunities are concentrated within the destination class. However, downwardly mobile individuals may resist identifying with their new class position and, consequently, show limited willingness to form strong ties with destination-class contacts. This ambivalence may result in relative social isolation or in the maintenance of weak, superficial ties across multiple contexts, producing networks that are relatively diverse yet lack full integration into any single class milieu.

Whether individuals experiencing upward or downward social mobility modify their personal networks toward greater or lesser homogeneity may depend substantially on the temporality and characteristics of trajectory changes. Maintaining class heterogeneous networks requires considerable effort, including cultural translation, navigation between different codes, and additional emotional investment (Erickson, 1996). With limited time and cognitive resources, mobile individuals may rationalize their network ties (Mollenhorst et al., 2014). Consequently, over the long term, mobile individuals might tend to homogenize their networks toward the destination class, especially if mobility is upward and becomes consolidated. If this occurs, initial heterogeneity may represent a transitional phase that eventually resolves toward homogeneity as individuals become more firmly established in their new class position. Additionally, the distance of mobility—that is, the extent of class displacement—could matter significantly. Indeed, short-distance movements within the class structure may produce less network disruption than long-distance movements (Goldthorpe and Jackson, 2007). Similarly, episodic instability versus clear, sustained trajectories may generate different network dynamics. Individuals experiencing brief or unstable class transitions may lack sufficient time or motivation to reconfigure their networks, whereas those following clear, sustained mobility paths may more thoroughly transform their social circles (Friedman, 2016). This implies that mobility-induced network heterogeneity may be conditional, temporary, and unevenly distributed across trajectories, rather than a permanent erosion of class-based closure.

Although some degree of intragenerational mobility is possible, most individuals remain stable in a specific class position throughout their lives. We posit that this stability likely reflects not merely the absence of mobility but rather consolidated class positions that facilitate cumulative homophily processes across the life course. Individuals who remain within the same class position over their career benefit from continuous exposure to structurally similar others within stable occupational contexts, residential environments, and associational settings. These sustained opportunity structures may enable the formation and maintenance of densely homogeneous networks through repeated interaction with class peers (Bourdieu, 1990). Moreover, class-stable individuals experience cultural continuity that reinforces affinity with same-class contacts: their consumption patterns, leisure preferences, normative orientations, and interaction styles remain aligned with their class position, facilitating natural rapport and sustained relationships with class peers (Lizardo, 2006; Jarness, 2017). Unlike mobile individuals who must navigate cultural transitions and potentially incompatible social codes, stable individuals move through coherent class-specific class habitus where shared understandings and practices make same-class relationships easier to initiate and maintain. Consequently, class stability provides both structural and cultural conditions that promote network homogeneity through uninterrupted accumulation of same-class ties over time.

Nevertheless, the homogeneity-generating effects of class stability may vary systematically across class positions, reflecting the distinct mechanisms through which different classes maintain network boundaries. Upper-class stability likely produces the most pronounced network homogeneity, as prolonged tenure in upper class positions provides continuous access to exclusive institutional spaces—elite workplaces, prestigious social clubs, affluent neighborhoods—that actively engineer same-class interaction while screening out cross-class contact (Khan, 2011; Lareau, 2011). Similarly, lower-class stability may generate substantial network homogeneity, although through fundamentally different mechanisms, including sustained exposure to spatially concentrated disadvantage, residential segregation, and limited institutional access that constrain contact pools to same-class others (Espinoza and Otero, 2024). For lower-class stable individuals, homogeneity reflects structural exclusion rather than strategic boundary maintenance, yet the cumulative effect of remaining in disadvantaged positions over time is similarly homogeneous network configurations (Espinoza, 1999).

In contrast, middle-class stability may produce more moderate levels of network homogeneity through two distinct mechanisms. First, middle-class positions occupy a contradictory location within class relations, simultaneously exercising authority over working-class positions while remaining subordinate to the upper class, at least in the workplace (Wright, 1997). Under conditions of stability, this dual positioning likely reinforces functionally distinct relational ties with both ends of the hierarchy as a structural by-product of their location in the relations of production (Wright and Cho, 1992). Second, middle-class individuals lack both the institutional resources for active boundary maintenance available to the upper class and the compulsion of structural confinement characteristic of the working class, making it more likely that they inhabit residentially mixed environments and participate in varied associational settings that facilitate cross-class contact (Jarness, 2017; Friedman, 2016). Without either the capacity for active closure or the pressure of structural confinement, middle-class networks are likely to exhibit lower homogeneity by default. Taken together, these mechanisms suggest that stable middle-class individuals may occupy more diverse relational positions within the class structure, not necessarily through a deliberate bridging role or chosen network strategy, but through the structural characteristics and dual relational embeddedness of middle-class social locations themselves (e.g., Otero et al., 2021).

### Hypotheses

Our theoretical considerations lead to several empirical expectations about the relationship between intragenerational mobility and class-based network homogeneity.

First, we expect that individuals who remain stable in their class position throughout their working lives will exhibit higher levels of class homogeneity in their personal networks compared to mobile individuals (H1). This considers the potential cumulative effects of sustained exposure to same-class contacts within coherent occupational, residential, and associational contexts, combined with cultural continuity that facilitates natural affinity with class peers. In contrast, mobile individuals, whether moving upward or downward, experience trajectory-induced disruptions that can generate more heterogeneous network configurations.

Second, although mobile individuals can exhibit lower class homogeneity than stable individuals, we expect downwardly mobile individuals to display higher levels of homogeneity than upwardly mobile individuals (H2). This distinction arises because downward mobility may combine shame-induced rupture of origin-class ties, economic constraints limiting participation in origin-class spaces, and resistance to identifying with the destination class, paradoxically producing more circumscribed networks. In contrast, upwardly mobile individuals may more successfully maintain dual connections, possessing greater resources to sustain origin-class ties, particularly family relationships and long-standing friendships, while accessing destination-class networks through new occupational and residential contexts, accompanied by aspirational motivations for status consolidation, thereby likely creating more heterogeneous bridging structures.

Third, we anticipate that the homogeneity-generating effects of stability may vary systematically across class positions (H3). Specifically, stability in upper-class and lower-class positions should produce the most pronounced network homogeneity, in the former case through exclusive institutional access and strategic boundary maintenance; in the latter through structural exclusion and spatial concentration. In contrast, middle-class stability may generate more moderate homogeneity levels, as middle-class positions inherently involve more diverse interaction contexts that facilitate cross-class contact even in the absence of mobility.

Finally, we consider that long-distance mobility may produce stronger effects on network composition than short-distance movements (H4), as greater class displacement provides both increased opportunities and stronger motivations for network transformation, potentially intensifying both the disruption of origin-class ties and the formation of destination-class relationships.

## Data, variables and methods

### Data

We use data from the Chilean Longitudinal Social Survey (ELSOC), designed and implemented by the Centre for Social Conflict and Cohesion Studies (COES). ELSOC is a nationally representative panel survey of the urban adult population (aged 18–75) that follows individuals across seven waves collected between 2016 and 2023 (COES, 2024). For the present study, we draw on four waves—2016, 2018, 2021, and 2023—which together form a weighted, unbalanced panel. The original sample included 2,927 respondents in Wave 1, 2,229 in Wave 2, 1,739 in Wave 3, and 1,737 in Wave 4. Despite gradual attrition, the longitudinal structure maintains robust representativeness of the urban population through calibrated panel weights provided by COES. This longitudinal design provides an exceptional opportunity to examine changes in class segregation within personal networks, while accounting for social mobility and individual heterogeneity (Otero et al., 2021; Plaza et al., 2026).^1^

### Dependent variables

To measure class homogeneity, we use the position generator instrument included in ELSOC, which asks respondents how many people they know in 13 occupations of varying occupational status: manager of a large firm, secretary, accountant, lawyer, doctor, preschool teacher, shop assistant, waiter, taxi driver, car mechanic, street vendor, office cleaner, and university professor. These occupations are classified into three broad class categories: upper, middle, and lower class positions aligned with different class schemas. In the case of the Oesch schema (Oesch, 2006), for example, occupations derived from the position generator can be aggregated into upper-middle class (professional and managerial occupations), lower-middle class (technical and administrative jobs), and working-class positions (manual and service-sector occupations) (Lindh and Andersson, 2025).

We calculate the proportion of social ties belonging to their own class, thereby generating an indicator of class-based network segregation. Specifically, for each respondent *i*, three variables were first computed to capture the proportion of occupations in which they reported knowing someone belonging to the upper-middle (Uᵢ), lower-middle (Lᵢ), and working class (Wᵢ). These values represent the relative distribution of social contacts across class strata, with higher proportions indicating greater relational reach into that class.

Building on these measures, a synthetic index of class homogeneity (CHᵢ) was constructed to capture the share of known occupations corresponding to the respondent’s own class position (Cᵢ), defined as:


$$CH_{i}=\{\begin{array}{l} \frac{U_{i}}{U_{i}+L_{i}+W_{i}\;},\text{if}\;C_{i}=\text{Upper}-\text{middle Class}\\ \frac{L_{i}}{U_{i}+L_{i}+W_{i}\;},\text{if}\;C_{i}=\text{Lower}-\text{middle Class}\\ \frac{W_{i}}{U_{i}+L_{i}+W_{i}\;},\text{if}\;C_{i}=\text{Working Class} \end{array}$$

The index ranges between 0 and 1, where higher values denote greater within-class closure, i. e., networks composed mainly of ties to people from the same social class, and lower values reflect more heterogeneous, cross-class networks. Conceptually, this indicator operationalizes the degree of relational segregation that characterizes individual social worlds. It thus provides a consistent and comparable measure of the permeability of class boundaries in Chilean personal networks over time (Otero et al., 2021).

This operationalization also mirrors the structure proposed by Iturra-Sanhueza (2026) for the ISSP but is specifically adapted to the Chilean occupational context of ELSOC. By distinguishing between relational ties toward different class strata and summarizing them into a unified measure of closure, the variable captures both the openness and closure dimensions of social class relations, two mechanisms that together shape the reproduction or erosion of class boundaries in stratified societies.

### Independent variables

The main explanatory variables capture individual social class and intragenerational social mobility. Class position is operationalized using an adapted version of the Oesch (2006) class schema, which combines occupational skill level, employment relations, and work orientation. In the Chilean context, we group respondents into three aggregated categories: upper-middle class (professionals, managers, and higher-grade technicians), lower-middle class (routine non-manual, clerical, and lower technical occupations), and working class (manual and service-sector workers).

To capture short-term or conjunctural mobility, we compare respondents’ class position between consecutive survey waves (2016–2018, 2018–2021, and 2021–2023). This measure reflects temporary positional changes resulting from transitions in employment status, job promotions, or downward occupational moves. Such changes are analytically relevant because they indicate the fluid component of social stratification, movements that may temporarily expand or contract individuals’ exposure to other social groups. In relational terms, conjunctural mobility captures the immediate reconfiguration of personal networks following occupational change, when individuals’ social environments tend to diversify before eventually stabilizing. Hence, it represents short-term opportunities for cross-class interaction and a test of the adaptability of network boundaries to life-course contingencies.

Structural mobility is defined as the respondent’s class position at each wave relative to their baseline position in 2016. This indicator focuses on the direction and persistence of change over time, distinguishing whether individuals have experienced an upward, downward, or stable trajectory during the entire study period. Conceptually, structural mobility expresses the degree of permeability in the class structure itself, showing whether individuals who start in lower or higher social positions manage to cross class boundaries and sustain that transition. From a sociological standpoint, this type of mobility reveals the structural underpinnings of network segregation: if class barriers are rigid, networks will remain highly homogeneous even when occupational movement occurs; if boundaries are permeable, sustained mobility should correspond to greater relational diversity (Blau and Schwartz, 1984).

Finally, we construct a measure of cumulative trajectory, which synthesizes respondents’ longitudinal class paths across all four waves, distinguishing between stable, upwardly mobile, downwardly mobile, and oscillating patterns. This cumulative indicator captures long-term sequences of movement and stability, integrating both direction and frequency of class transitions. The underlying rationale is that repeated movement—either upward or downward—can generate lasting changes in social composition by expanding individuals’ relational fields and reshaping their class identity (Bourdieu, 1984; Lin, 2001). By contrast, stability or repeated oscillation around a given class position is expected to reinforce habitual social boundaries, producing stronger within-class closure. Thus, cumulative mobility encapsulates how class trajectories unfold over time and how these trajectories structure the persistence or transformation of relational segregation.

### Control variables

To mitigate the risk of omitted-variable bias, all models include a set of sociodemographic and socioeconomic controls that are theoretically and empirically associated with patterns of personal network formation and class-based social closure (DiPrete et al., 2011; Hofstra et al., 2017; Otero et al., 2021). Specifically, we control for age, sex, education, household income, employment status, occupational prestige, and network size. These factors capture structural inequalities that shape individuals’ opportunities for interaction and the likelihood of forming class-homogeneous ties.

Age is measured in years and treated as a continuous variable. Older individuals typically exhibit higher network stability and denser intra-class connections, while younger respondents tend to engage in more fluid, transitional social settings. Sex is coded as a binary variable (1 = female, 0 = male), acknowledging the persistent gendered division of social and occupational spheres. Prior research indicates that women’s networks tend to be smaller but more multiplex and family-oriented, whereas men’s networks are often broader and oriented toward occupational contexts (McPherson et al., 2001).

Education is measured as the number of completed years of formal schooling. Higher educational attainment tends to broaden individuals’ social horizons, providing greater opportunities for cross-class exposure through professional and institutional settings. Monthly household income, measured in thousands of Chilean pesos and log-transformed to reduce skewness, serves as a proxy for access to material and cultural resources that facilitate participation in heterogeneous social environments. Employment status distinguishes between respondents who are currently employed and those who are not, capturing the central role of workplaces in generating both class-similar and cross-class social ties.

Lastly, we include a measure of network size, calculated as the total number of acquaintances reported across the 13 occupations included in the position generator. Rather than relying on indirect estimation methods such as the Network Scale-Up Method (Killworth et al., 1998), this operationalization directly reflects respondents’ self-reported scope of social connections (Otero et al., 2021). Larger networks are likely to entail more opportunities for heterophilous contact, while smaller networks tend to reinforce class-based closure. Including this variable allows us to isolate the specific influence of class and social mobility from general differences in network extensiveness.

## Methods

We estimate a series of longitudinal and cross-sectional models designed to assess how social class, different forms of intragenerational mobility, and long-term class trajectories shape the degree of class homogeneity in personal networks. Given the nested structure of the ELSOC panel, with repeated observations clustered within individuals, we rely on hybrid random-effects models that partition within- and between-person variation (Allison, 2009; Bell and Jones, 2015).^2^ This modelling strategy, often referred to as Random-Effects Within-Between (REWB), allows us to examine how changes in individuals’ social circumstances across waves influence their network composition, while simultaneously estimating stable differences between individuals. Following best practices for hybrid models (Enders and Tofighi, 2007), all time-varying continuous variables, i.e., income and network size, are decomposed into person-specific means (between effects) and deviations from these means (within effects). This specification avoids conflating structural differences between individuals with short-term fluctuations across waves.

Our analytical approach proceeds in three steps. First, we estimate a baseline REWB model that includes class of origin alongside sociodemographic controls and the between/within components of income and network size. Second, we incorporate two alternative operationalizations of social mobility. The first captures inter-wave or conjunctural mobility, defined as upward, downward, or stable transitions between consecutive waves (2016–2018, 2018–2021, 2021–2023). Because this indicator necessarily requires information from the previous wave, the earliest year included in these models is 2018. The second mobility indicator measures position relative to class origin, distinguishing whether respondents are above, below, or equal to their initial 2016 class status. This relative measure approximates medium-term structural shifts and provides a robustness check against immediate, conjunctural moves. Third, we estimate a cross-sectional model focusing on cumulative class trajectories. This variable classifies individuals into stable, upwardly mobile, downwardly mobile, or oscillating patterns after observing their full sequence of class positions between 2016 and 2023. Because trajectory categories are inherently ex post constructs, using them to predict outcomes observed before 2023 would risk post-treatment bias. To preserve temporal coherence, we therefore restrict this final model to the 2023 wave and estimate a weighted OLS model rather than a multilevel model (since each individual contributes only one observation). For this model, structural indicators of income and network size are computed as person-specific averages across the full 2016–2023 period, capturing respondents’ longer-term socioeconomic standing.

The analytical sample is based on an unbalanced panel of 2,520 individuals observed between 2016 and 2023 (6,400 person-wave observations). For the longitudinal models (A and B), the sample is restricted to observations with valid inter-wave mobility, defined from 2018 onwards, resulting in 3,880 observations from 1,906 individuals. To ensure comparability across specifications, we further restrict the sample to complete cases on all variables included in the full REWB model, yielding a final analytical sample of 3,735 observations from 1,874 individuals. The cumulative trajectory model (C) is estimated cross-sectionally using only the 2023 wave, with a final sample of 1,114 individuals after applying the same complete-case restriction.

Across all models, panel weights provided by COES are applied to ensure representativeness of the urban population. Goodness-of-fit metrics (AIC, BIC, log-likelihood) and the number of observations and clusters are reported to facilitate comparison across specifications. Together, this modelling strategy allows us to disentangle episodic mobility from persistent class differences and long-term trajectories, providing a comprehensive account of how class structure and life-course movement shape the relational boundaries of social class in Chilean society.^3^

## Results

### Descriptive analyses

We begin by examining descriptive patterns in class homogeneity and network composition across the four waves of ELSOC (2016–2023). Table A1 (see Supplementary material) shows that class homogeneity remains relatively stable over time, with proportions ranging from 0.403 to 0.454 across the period. The highest value appears in 2016 (0.454, SD = 0.245), followed by a decline in 2018 (0.434, SD = 0.224) and 2021 (0.403, SD = 0.184), and a partial rebound in 2023 (0.432, SD = 0.192). These fluctuations suggest moderate change but no structural transformation in the degree of class-based homogeneity in the Chilean adult population, which are consistently near the midpoint of the scale, indicating that even though they are far from fully segregated, they remain significantly patterned by class.

**Figure 1:**
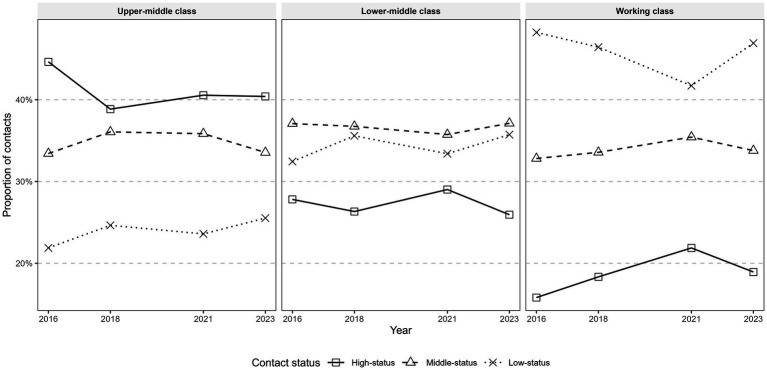
Network composition by respondent’s class position. ELSOC survey 2016–2023. Weighted estimates.

To better understand the compositional structure of these networks, Figure 1 shows network ties by occupational status over time, revealing three distinctive patterns. First, both upper-middle-class and working-class respondents exhibit similarly high proportions of same-class contacts, consistently just above 40%. This symmetry indicates that network closure characterizes interaction at both extremes of the chilean stratification structure (Otero et al., 2021). Second, lower-middle class individuals depart from this pattern: their share of same-class contacts is substantively lower (around 27–29%), and their networks contain a balanced proportion of high, middle and low status contacts, positioning them as a relational bridge between extremes. Lastly, middle-status contacts account for a remarkably stable share of network composition across all respondent classes and survey waves, suggesting that the fundamental architecture of class-based networks is resilient to the moderate patterns of mobility in the sample.

**Figure 2:**
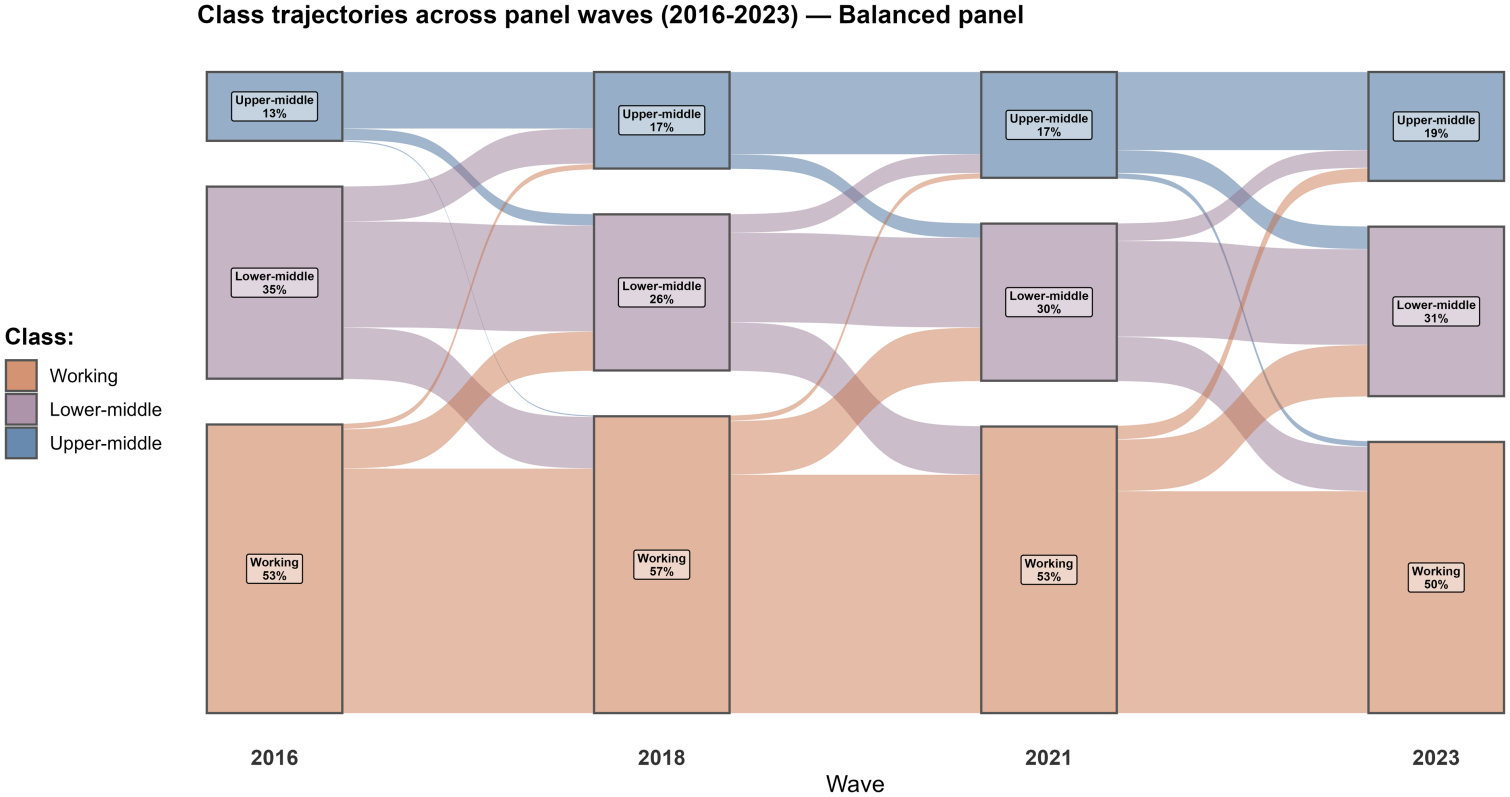
Class trajectories across panel waves (balanced panel). ELSOC survey 2016–2023. Weighted estimates.

Figure 2 traces class trajectories across all four waves, revealing the central patterns in the mobility structure of the balanced sample using attrition-corrected weights. Figure A1 (see Supplementary material) presents the equivalent diagram for the full unbalanced sample, where gradual growth of upper-middle class representation reflects selective attrition rather than genuine structural upward mobility (from 12 to 19%, see Table A1). Despite this observation, several patterns emerge in the mobility structure of both samples. A substantive percentage of respondents remain stable in their class position throughout the study period, confirming that class stability is the dominant pattern between waves. Among those who do move, upward transitions are modestly more frequent than downward ones. Finally, mobility flows are heavily concentrated in transitions between working and lower-middle class positions, compared to upper-middle class movement between waves. This asymmetry reinforces the bridging role of lower-middle class identified in Figure 1. It is not only relationally open, but also the primary site of structural fluidity, absorbing and releasing individuals from both directions across the panel.

**Figure 3:**
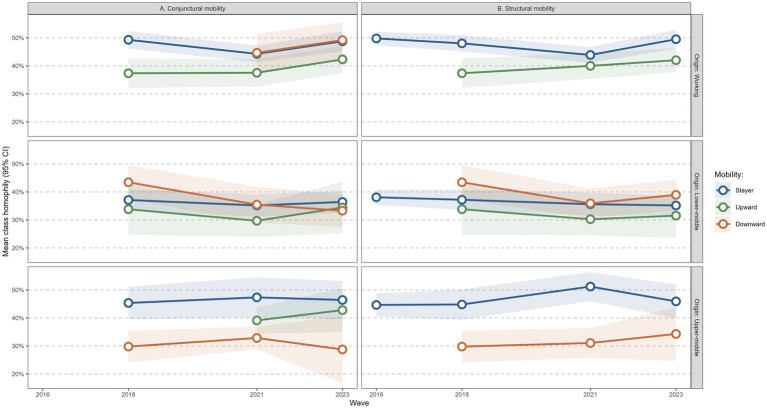
Homogeneity means by origin class, mobility type (conjunctural and structural) and wave. Source: ELSOC 2016–2023. Weighted estimates. Panel A excludes wave 2016.

Lastly, Figure 3 compares these fluidity tendencies to network patterns by examining class-based network homogeneity across mobility types and class of origin. Stayers consistently display higher homogeneity than mobile individuals across all origin classes and waves, confirming the robustness of the stability-homogeneity association. The gap is largest among upper-middle class origins, where downwardly mobile individuals falls to approximately 30%, suggesting that falling from a higher-class position ruptures origin-class networks, although this pattern must be interpreted cautiously given the comparatively small proportion of downwardly mobile upper-middle-class respondents. Most theoretically consequential, the lower-middle class shows virtually no differentiation by mobility type, with homogeneity levels tightly between 30 and 38%, confirming that the relational openness in this stratum is structural rather than mobility-induced.

### Explanatory analyses

Since our descriptive patterns suggest that changes in class position may alter the composition and homogeneity of personal networks, it is necessary to evaluate whether social mobility actually produces shifts in relational closure over time. To do so, in this section, we examine how class of origin, intragenerational mobility, and cumulative class trajectories shape the degree of class homogeneity in personal networks. Table 1 presents three multivariate models that combine between-person structural differences with within-person changes across waves. Model 1 evaluates inter-wave mobility; Model 2 assesses mobility relative to origin; and Model 3 employs cumulative trajectories using cross-sectional data from 2023. Together, these specifications provide a comprehensive assessment of whether short-term positional changes, medium-term structural displacement, and long-term biographical patterns account for variation in class-based network homogeneity.

**Table 1:** Regression models predicting class-based homogeneity by intragenerational mobility.

Predictors	Model 1	Model 2	Model 3
	Inter-wave mobility	Mobility from origin	Trajectories (2023)
Intercept	0.516***	0.514***	0.526***
	(0.015)	(0.015)	(0.020)
Class origin: lower-middle (ref. working)	−0.051***	−0.052***	
	(0.012)	(0.013)	
Class origin: upper-middle	−0.023	−0.037	
	(0.020)	(0.021)	
Inter-wave mobility: upward (ref. stayer)	−0.049***		
	(0.008)		
Inter-wave mobility: downward	−0.010		
	(0.008)		
Mobility from origin: upward (ref. stayer)		−0.067***	
		(0.008)	
Mobility from origin: downward		−0.011	
		(0.011)	
Trajectory: stable lower-middle class (ref. stable working class)			−0.106***
			(0.025)
Trajectory: stable upper-middle class			0.011
			(0.033)
Trajectory: only upward			−0.053**
			(0.017)
Trajectory: only downward			−0.079***
			(0.020)
Trajectory: oscillating			−0.073***
			(0.017)
Network size (within)	0.000	0.000	
	(0.000)	(0.000)	
Income (within)	−0.000	−0.000	
	(0.000)	(0.000)	
Network size (between)	−0.000	−0.000	−0.000
	(0.000)	(0.000)	(0.000)
Income (between)	0.000 *	0.000 *	0.000 *
	(0.000)	(0.000)	(0.000)
Woman	−0.016	−0.014	−0.023 *
	(0.010)	(0.010)	(0.011)
Education: secondary (ref. primary)	−0.054***	−0.053***	−0.030
	(0.013)	(0.013)	(0.020)
Education: vocational	−0.097***	−0.091***	−0.095***
	(0.015)	(0.015)	(0.024)
Education: tertiary	−0.078***	−0.073***	−0.090***
	(0.016)	(0.016)	(0.024)
Year: 2021 (vs baseline)	−0.019**	−0.016**	
	(0.006)	(0.006)	
Year: 2023 (vs baseline)	0.007	0.011	
	(0.006)	(0.006)	
AIC	2431.244	2408.113	1134.183
BIC	2537.077	2513.947	1199.388
Log-likelihood	−1198.622	−1187.057	−554.092
Num. obs.	3,735	3,735	1,114
Num. groups (id)	1874	1874	

ELSOC Survey 2016–2023. Weighted figures. ***p < 0.001, **p < 0.01, *p < 0.05.

Model 1 indicates that social class of origin exerts a strong and persistent structural influence on class homogeneity in personal networks. Respondents from the lower-middle class exhibit significantly lower levels of homogeneity than working-class individuals (β = −0.051, SE = 0.012, *p* < 0.001), while those from the upper-middle class show no statistically significant difference (β = −0.023, SE = 0.020, *p* = 0.248). Turning to conjunctural mobility, upward moves between waves predict substantially reduced class-based network homogeneity relative to stayers (β = −0.049, SE = 0.008, *p* < 0.001), whereas downward moves show no detectable effect (β = −0.010, SE = 0.008, *p* = 0.215). These results suggest that upward transitions temporarily diversify networks, whereas downward transitions do not disrupt class closure to the same extent.

Model 2, which measures class position relative to origin, yields consistent patterns. Lower-middle-class origin remains significantly associated with lower homophily (β = −0.052, SE = 0.013, *p* < 0.001), and upper-middle-class origin again shows no significant effect (β = −0.037, SE = 0.021, *p* = 0.084). Relative upward mobility from origin is negatively associated with homophily (β = −0.067, SE = 0.008, *p* < 0.001), whereas relative downward mobility has no statistical impact (β = −0.011, SE = 0.011, *p* = 0.316). These findings reinforce the interpretation that sustained improvement in class position expands relational diversity, while downward moves are less socially disruptive.

Finally, Model 3 evaluates cumulative class trajectories using the 2023 cross-sectional data. Relative to individuals stably positioned in the working class, those stably in the lower-middle class exhibit markedly lower levels of class homogeneity in personal networks (β = −0.106, SE = 0.025, *p* < 0.001), whereas stable upper-middle-class individuals do not differ significantly (β = 0.011, SE = 0.033, *p* = 0.741). Persistently upwardly mobile respondents also display reduced homophily (β = −0.053, SE = 0.017, *p* = 0.002), as do those following oscillating trajectories (β = −0.073, SE = 0.017, *p* < 0.001). Downwardly mobile individuals show even larger reductions (β = −0.079, SE = 0.020, *p* < 0.001). Together, these trajectory effects indicate that long-term intragenerational class mobility is associated with more open and heterogeneous personal networks, whereas stable class positions tend to reinforce class homogeneity.

**Figure 4:**
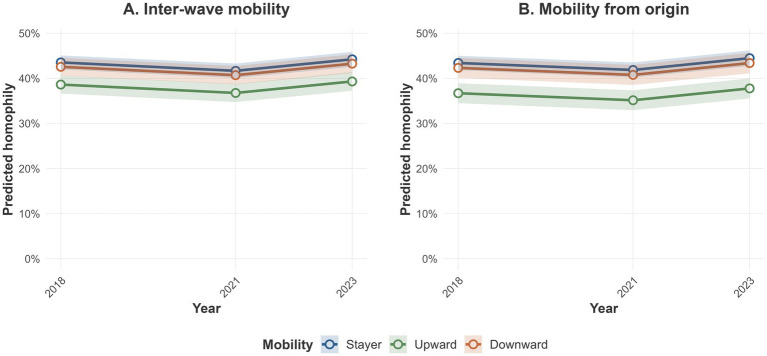
Predicted class-based homogeneity in personal networks by social mobility. ELSOC survey 2016–2023.

To complement the multivariate estimates presented above, Figure 4 displays predicted levels of class-based homogeneity in personal networks derived from Models 1 and 2, allowing for a clearer visualization of how different forms of social mobility shape class homophily. Panel A reveals consistent and pronounced gradients across categories of inter-wave mobility. Individuals who remain in the same class exhibit the highest predicted class-based network homogeneity (approximately 43–45%), whereas upwardly mobile respondents display substantially lower levels (approximately 37–39%), indicating that upward transitions are associated with more heterogeneous networks. Downwardly mobile individuals occupy an intermediate position but remain closer to stayers, suggesting more limited relational disruption. Across all groups, class homogeneity declines in 2021 and then recovers in 2023, a pattern consistent with pandemic-related instability in network composition.

Panel B extends this analysis using mobility relative to class origin. The pattern closely mirrors the inter-wave results: upwardly mobile individuals again display the lowest class homogeneity in personal networks (≈36–38%), while those who remain at their origin class maintain the highest values (≈43–45%). Downward mobility produces only modest declines relative to stayers, reinforcing that upward transitions are the primary force associated with network diversification. The stability of these differences across time points indicates that both short-term and medium-term upward mobility systematically widen individuals’ relational fields, whereas stable or downward trajectories preserve higher within-class closure. Together, the two panels provide a coherent picture of the relational consequences of social mobility under a stratified class structure.

**Figure 5:**
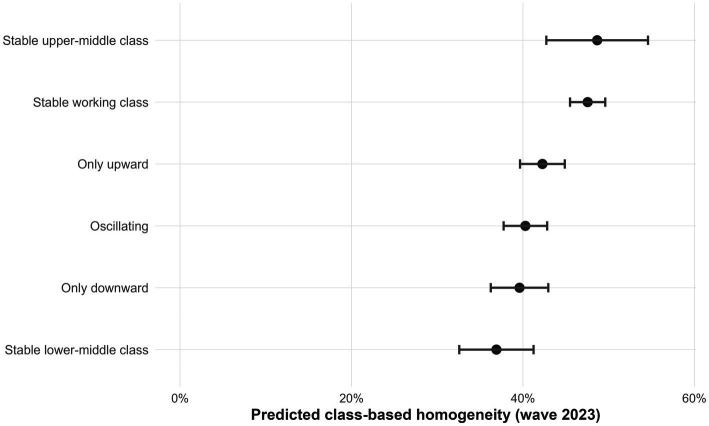
Predicted class-based homogeneity in personal networks by cumulative trajectory. ELSOC Survey 2016–2023.

Building on these temporal models, Figure 5 presents predicted class-based network homogeneity in personal networks for long-term cumulative class trajectories, offering a biographical perspective on how enduring mobility patterns shape social networks. These predictions are based on the cross-sectional weighted model of wave 2023; that is, Model 3. Individuals who remain stable in the working or upper-middle class show the highest predicted class homogeneity (≈45–48%), supporting the idea that long-term positional stability reinforces class-bounded relations. In contrast, respondents stably located in the lower-middle class exhibit significantly lower homophily (≈38–40%), consistent with this group’s more permeable position within Chile’s class structure.

Cumulative mobility patterns further illustrate how extended movement reshapes relational boundaries. Those who experience only upward mobility have lower homophily (≈39–41%), while individuals with exclusively downward trajectories show even greater heterogeneity (≈36–38%). Oscillating respondents, who alternate between class positions across waves, also display comparatively low homophily, suggesting that repeated positional shifts prevent the consolidation of class-bounded networks. Overall, Figure 5 demonstrates that long-term class mobility, whether upward, downward, or oscillatory, corresponds to more diverse social networks, whereas stable class locations continue to generate and reproduce strong within-class closure.

### Additional analyses

To assess the robustness of our findings and to situate our class homogeneity index (CHi) in relation to other commonly used metrics in the network literature, we replicated all main analyses using four alternative measures of network diversity: Shannon’s entropy, Blau’s index, normalized Blau’s index, and the Herfindahl–Hirschman Index (HHI). The first three are diversity metrics in which higher values indicate greater diversity across contacts, while the HHI is a concentration metric in which higher values indicate greater homogeneity. Tables A2–A4 (in the Supplementary material) present the results for each set of models.

Regarding inter-wave mobility (Table A2), results across all four alternative metrics are consistent with the main findings. Both upward and downward inter-wave mobility are associated with significantly greater network diversity compared to class-stable individuals, with upward mobility showing a stronger effect across all metrics (Shannon: β = 0.099, *p* < 0.001; Blau: β = 0.026, p < 0.001) than downward mobility (Shannon: β = 0.057, *p* < 0.001; Blau: β = 0.014, *p* < 0.05). It is worth noting that while downward mobility was not statistically significant in the main CHi models, it does reach significance across all diversity metrics, suggesting that any form of class movement, whether upward or downward, is associated with greater network diversity relative to class stability.

Turning to mobility from class of origin (Table A3), results again replicate the main patterns. Upward mobility from origin is significantly associated with greater network diversity across all metrics (Shannon: β = 0.056, *p* < 0.001; Blau: β = 0.015, *p* < 0.01), while downward mobility from origin shows no statistically significant effect in any of the alternative specifications, a finding that reinforces our interpretation of asymmetric mobility effects on network composition.

With respect to cumulative class trajectories (Table A4), the alternative metrics confirm that oscillating trajectories show the strongest association with network diversity (Shannon: β = 0.184, *p* < 0.001; Blau: β = 0.055, *p* < 0.001), followed by consistently upward trajectories (Shannon: β = 0.104, *p* < 0.01; Blau: β = 0.027, *p* < 0.05), both relative to stable working-class individuals. Stable lower-middle-class and stable upper-middle-class trajectories do not reach statistical significance in any of the diversity metrics, suggesting that diversity metrics are less sensitive to the relational dynamics of stable class positions, a pattern which underscores the analytical value of the CHi for capturing class-specific closure processes.

Taken together, these robustness checks confirm that the substantive conclusions of the study hold across alternative operationalizations of network composition. However, this exercise also allows us to clarify an important analytical distinction. While class homogeneity and diversity metrics are negatively correlated, as would be expected, these correlations are notably low. This suggests that the CHi captures a conceptually distinct dimension of network composition associated with class closure processes, one that is not equivalent to what diversity metrics measure (Abascal and Baldassarri, 2015). In other words, anchoring network composition to class boundaries reflects dynamics of social closure that go beyond mere diversity or concentration of ties, and the two types of metrics should not be conflated.

### Summary

In summary, our empirical findings provide varying levels of support for the four hypotheses proposed. Hypothesis 1, which predicted that class-stable individuals would exhibit higher network homogeneity than mobile individuals, receives strong and consistent support across all model specifications. Regardless of whether we examine inter-wave transitions, movement relative to origin, or cumulative trajectories, individuals remaining in the same class position throughout the study period consistently display substantially more class-bounded networks than those experiencing positional changes. This pattern held across all waves and proved robust to different operationalizations of both stability and mobility, providing clear empirical confirmation of the cumulative homophily effects generated by sustained class positions.

Hypothesis 2, which anticipated that downwardly mobile individuals would display higher homogeneity than upwardly mobile individuals, receives partial support. Models 1 and 2 demonstrate that upwardly mobile individuals exhibit substantially lower homogeneity than both stable and downwardly mobile respondents, with downwardly mobile individuals showing levels closer to stable individuals. However, Model 3, examining cumulative trajectories in 2023, reveals less pronounced differences between upward and downward mobility patterns, with both types of sustained movement associated with reduced homogeneity relative to stability. This suggests that the hypothesized asymmetry between upward and downward mobility holds more clearly for short-term and medium-term transitions than for long-term cumulative patterns, warranting interpretation as partial rather than complete support.

Hypothesis 3, predicting that stability effects would vary systematically across class positions, receives support. We found that stable middle-class individuals exhibit significantly lower homogeneity than stable upper-middle-class and working-class respondents, consistent with the theoretical argument that middle-class positions involve both a contradictory relational location within class relations and a relative absence of closure mechanisms, producing network diversity through structural rather than deliberate means.

Finally, Hypothesis 4, which expected long-distance mobility to produce stronger effects than short-distance movements, receives support through our cumulative trajectory analysis. Long-term mobility patterns—whether upward, downward, or oscillatory—all correspond to substantially more diverse social networks compared to stable class locations, demonstrating that sustained movement through the class structure produces more profound relational transformations than isolated positional changes. These findings are discussed in detail in the final section of the paper.

## Conclusions and discussion

This study examined how class-based network homogeneity evolves over time and whether intragenerational social mobility reshapes personal network composition in Chile. Drawing on four waves of longitudinal data from ELSOC (2016–2023), we assessed the extent to which class stability and different forms of social mobility, such as conjunctural, structural, and cumulative, influence the degree of class homogeneity in personal networks. Our analysis contributes to existing research by providing empirical evidence on the temporal dynamics of class-based network segregation, a dimension that has remained largely unexplored due to the scarcity of longitudinal datasets simultaneously capturing socioeconomic positioning and detailed network configurations. Based on our results, we are able to highlight and discuss several important findings.

First, our descriptive findings revealed important patterns regarding the overall structure of class-based networks in Chile. On the one hand, we observed that class homogeneity levels remained relatively stable across the 2016–2023 period, fluctuating between 40 and 45% without evidence of structural transformation. This stability suggests that despite economic and social changes occurring during this period, the fundamental mechanisms generating class-based network segregation—structural constraints on meeting opportunities and cultural preferences shaped by class position—continue operating with consistent intensity (Blau and Schwartz, 1984; Bourdieu, 1984). On the other hand, both upper-middle-class and working-class respondents exhibited similarly high proportions of same-class contacts across all waves, with levels consistently above 40%. This symmetry at opposite ends of the class hierarchy indicates that network closure is not confined to privileged groups but rather characterizes social interaction at both extremes of the stratification structure (Otero et al., 2021). Moreover, the pervasive presence of middle-status contacts across all respondent classes, typically ranging between 32 and 37%, underscores the structural importance of the (lower-) middle class within Chile’s relational landscape, functioning as a bridge between higher-status and lower-status groups.

Second, our findings reveal that class stability is consistently associated with higher levels of network homogeneity compared to intragenerational social mobility. Individuals who remain in the same class position throughout the study period exhibit substantially more class homogeneity than those experiencing positional changes. This pattern holds across different operationalizations of mobility—inter-wave transitions, movement relative to origin, and cumulative trajectories—indicating that the relationship between stability and homogeneity is robust rather than contingent on measurement approach. These results align with theoretical expectations regarding the cumulative effects of sustained exposure to same-class contacts within coherent occupational, residential, and associational contexts (Bourdieu, 1984; Jarness, 2017). Class-stable individuals might benefit from continuous immersion in structurally similar environments where repeated interaction with class peers facilitates the formation and maintenance of densely homogeneous networks (Mollenhorst et al., 2014). Moreover, cultural continuity likely reinforces affinity with same-class contacts, as stable individuals may experience alignment in consumption patterns, leisure preferences, and interaction styles that make same-class relationships easier to initiate and sustain (Lizardo, 2006).

Third, we found that upwardly mobile individuals exhibit lower class homogeneity than downwardly mobile individuals, although this pattern is not consistent across all temporal specifications of mobility. The lower homogeneity observed among upwardly mobile individuals can be explained through multiple mechanisms operating simultaneously. On one hand, upward mobility exposes individuals to new occupational contexts, residential environments, and associational milieus that provide access to previously inaccessible social circles while often allowing preservation of connections to former class positions (Blau, 1977; Wegener, 1991). This dual network structure likely creates bridging configurations that span class boundaries, positioning upwardly mobile individuals between distinct social worlds (Lin, 2001; Burt, 2005). On the other hand, upwardly mobile individuals may possess both greater resources to sustain origin-class ties, particularly family relationships and long-standing friendships, and aspirational motivations for status consolidation that drive formation of destination-class networks (Friedman, 2016). The contrast with downwardly mobile individuals is particularly revealing. Downward mobility may precipitate rupture of prior relationships because they likely lack the economic and cultural resources required to sustain participation in origin-class spaces (Newman, 1988; Bourdieu, 1984). Furthermore, resistance to identifying with the destination class may limit formation of strong ties with new contacts, resulting in relative social isolation or maintenance of weak, superficial ties that do not fundamentally alter overall network homogeneity. Thus, while upward mobility opens pathways to network diversification through both structural opportunities and cultural aspirations, downward mobility appears to generate more circumscribed relational configurations.

Fourth, we found that stable lower-middle-class individuals exhibit significantly lower homogeneity than stable working-class respondents, whereas stable upper-middle-class individuals do not differ significantly from the working-class baseline. These findings suggest that middle-class positions inherently involve more diverse interaction contexts that facilitate cross-class contact even in the absence of mobility (Otero et al., 2021). The lower homogeneity observed among stable lower-middle-class respondents corresponds with their occupational composition—routine non-manual and clerical positions that occupy intermediate rungs within the class structure and provide exposure to both higher- and lower-status contacts through varied workplace settings and residential environments (Jarness, 2017). In contrast, the similarly high homogeneity levels observed among stable working-class and upper-middle-class individuals likely reflect fundamentally different mechanisms. For upper-class stability, homogeneity likely arises through exclusive institutional access and strategic boundary maintenance, as prolonged tenure provides continuous access to elite workplaces, prestigious social clubs, and affluent neighborhoods that actively engineer same-class interaction (Khan, 2011; Lareau, 2011). For lower-class stability, homogeneity likely stems from structural exclusion and spatial concentration, including sustained exposure to residential segregation and limited institutional access that constrain contact pools to same-class others (Massey, 1996). Thus, while both extremes exhibit comparable levels of network closure, the underlying processes may differ profoundly—strategic selection and resource hoarding at the top versus structural constraints and limited opportunities at the bottom (Bourdieu, 1999).

Finally, examining cumulative trajectories revealed that long-term mobility patterns produce particularly pronounced effects on network homogeneity. Individuals following persistently upward, downward, or oscillating trajectories all displayed substantially lower homogeneity than those remaining stable in working-class or upper-middle-class positions throughout the study period. These results indicate that, while mobility generally reduces homogeneity, differences between upward and downward trajectories are less consistent when mobility is considered cumulatively. These findings suggest that sustained or repeated mobility prevents the consolidation of class-bounded networks, as extended movement through the class structure continuously disrupts both structural opportunity contexts and cultural alignment mechanisms that generate homophily (McPherson et al., 2001; Feld, 1981). The particularly strong effects observed for oscillating trajectories are noteworthy, as repeated alternation between class positions appears to generate the most heterogeneous network configurations. This pattern may reflect the cumulative exposure to multiple class contexts across waves, which prevents full integration into any single class milieu while expanding the overall diversity of social contacts (Mollenhorst et al., 2014). Moreover, oscillating individuals may retain residual ties from each position they temporarily occupy, creating layered network structures that bridge multiple class boundaries simultaneously. These results underscore that the temporal dimension of mobility, not merely its direction, fundamentally shapes network composition, with sustained or repeated movement producing more profound relational transformations than isolated positional changes.

These findings contribute to broader theoretical debates regarding the relationship between social class and personal networks. Our results demonstrate that class boundaries, while permeable through mobility, remain consequential for structuring social relationships even in contexts of positional change. The persistence of elevated homogeneity among stable individuals and the relatively modest reductions observed among mobile respondents suggest that class-based network segregation reflects deeply embedded structural and cultural mechanisms that resist transformation through individual mobility alone (Blau and Schwartz, 1984; Lin, 2001). This resilience has important implications for understanding social cohesion and inequality reproduction. Class-homogeneous networks concentrate information, resources, and opportunities within class boundaries, potentially reinforcing existing inequalities and limiting cross-class understanding (Otero et al., 2022a). Mobile individuals, despite achieving positional advancement, may find their relational capital only partially transformed, retaining ties to origin contexts that complicate full integration into destination-class networks and cultural milieus (Friedman, 2016; Naudet, 2018).

Despite its contributions, our study has several limitations that must be acknowledged. First, while ELSOC provides exceptional longitudinal data, the limited number of waves restricts our capacity to distinguish between transient fluctuations and enduring changes in network composition. Some observed patterns, particularly the decline in homogeneity during 2021, may reflect pandemic-related disruptions rather than fundamental mobility effects, though subsequent recovery in 2023 suggests underlying stability. Second, the position generator instrument, while valuable for measuring cross-class ties, captures acquaintanceship rather than strong relationship ties, potentially underestimating the depth of class-based segregation in intimate networks. Third, selective attrition may bias our estimates if individuals who exit the panel differ systematically in their mobility patterns and network configurations from those who remain, though calibrated panel weights partially address this concern. Fourth, our occupational class schema aggregates heterogeneous positions within broad categories, potentially obscuring important within-class variation in network patterns.

These limitations point to important directions for future research. First, studies with longer panel designs and more waves would allow researchers to distinguish more rigorously between transient disruptions and structural transformations in class-based network composition, as well as to model non-linear trajectories of network change across the life course. Second, future work could complement position generator measures with instruments capturing strong ties and intimate networks, which may reveal deeper and more durable forms of class segregation that acquaintanceship-based measures cannot fully detect. Third, future research employing both finer-grained class schemas and more comprehensive position generators—covering a broader range of occupations within each class—would help clarify whether the patterns observed here hold across the internal fractions of each class or are driven by specific occupational subgroups. Finally, the growing availability of longitudinal network data globally offers new opportunities to assess the generalizability of these findings across different national and cultural contexts, and to identify the structural conditions—including welfare regime, labor market organization, and levels of residential segregation—under which mobility produces more or less profound network transformations. Chile represents an acute case of inequality and network segregation, making cross-national comparative research a particularly valuable next step to disentangle context-specific from more general dynamics linking class mobility and network composition.

Overall, our findings provide robust evidence that intragenerational social mobility reshapes class-based network homogeneity, though stable class positions continue generating substantial within-class closure. These patterns illuminate the relational dimensions of social stratification and mobility, demonstrating that class boundaries structure not only material opportunities but also the fundamental architecture of social connection. In this sense, class-based network segregation is not merely a reflection of individual preferences or voluntary association, but a structural outcome of stratification processes that systematically channel interaction within class boundaries. Reducing such segregation therefore requires more than expanding individual mobility chances. Indeed, it demands the creation of institutional contexts and opportunity structures that enable sustained cross-class contact, from integrated educational and residential environments to inclusive associational spaces that bring together individuals from different class positions. Ultimately, fostering more integrated social worlds requires not only reducing inequality in material terms, but also dismantling the structural arrangements that keep people from different class positions apart.

## Data Availability

Publicly available datasets were analyzed in this study. This data can be found at: https://dataverse.harvard.edu/dataverse/elsoc.

## Footnotes

1 For more information on the sampling design, visit the ELSOC website: https://coes.cl/elsoc/

2 This modeling strategy is particularly well suited to our research question, as it allows us to distinguish between differences across individuals and changes within individuals over time. In contrast to pooled OLS models, which conflate cross-sectional and longitudinal variation, the REWB approach separates between-person differences (e.g., individuals who are consistently more or less homogeneous) from within-person changes associated with mobility. At the same time, unlike fixed-effects models, which rely exclusively on within-individual variation and discard time-invariant differences, REWB models retain between-individual information while still providing unbiased estimates of within-person effects (Bell and Jones, 2015). This is particularly important in our case, as both stable class positions and mobility-related changes are theoretically meaningful and operate at different levels.

3 The code for all analyses described in this section is available from the authors upon request.
