# Supplementary material for: Social mobility and class segregation in personal networks over time: evidence from longitudinal data
Source: Front Sociol. 2026 Aug 3;11:1782127. doi: 10.3389/fsoc.2026.1782127 (PMC13477931; doi:10.3389/fsoc.2026.1782127)
Supplement: Supplementary file 1 [file Supplementary_file_1.docx]

**Supplementary Material**

| **Table A1.** Summary of Descriptive Statistics by Year (2016-2023). | | | | | | | | |
| --- | --- | --- | --- | --- | --- | --- | --- | --- |
|  | **2016** (*N* = 2,259) | |  |  | **2018** (*N =* 1,726) | |  |  |
| **Variable** | Mean | SD | Min | Max | Mean | SD | Min | Max |
| **Class Homogeneity** | |  |  |  |  |  |  |  |
| Class Homogeneity | 0.454 | 0.245 | 0 | 1 | 0.434 | 0.224 | 0 | 1 |
| **Occupational Class** | |  |  |  |  |  |  |  |
| Upper-Middle Class | 0.126 |  | 0 | 1 | 0.144 |  | 0 | 1 |
| Lower-Middle Class | 0.316 |  | 0 | 1 | 0.259 |  | 0 | 1 |
| Working Class | 0.558 |  | 0 | 1 | 0.596 |  | 0 | 1 |
| **Conjunctural Mobility** | |  |  |  |  |  |  |  |
| Upward |  |  |  |  | 0.147 |  | 0 | 1 |
| Downward |  |  |  |  | 0.125 |  | 0 | 1 |
| Stayers |  |  |  |  | 0.728 |  | 0 | 1 |
| **Structural Mobility** | |  |  |  |  |  |  |  |
| Upward |  |  |  |  | 0.134 |  | 0 | 1 |
| Downward |  |  |  |  | 0.114 |  | 0 | 1 |
| Stayers |  |  |  |  | 0.752 |  | 0 | 1 |
| **Cumulative Trajectory** | |  |  |  |  |  |  |  |
| Only Upward |  |  |  |  |  |  |  |  |
| Only Downward | |  |  |  |  |  |  |  |
| Stable |  |  |  |  |  |  |  |  |
| Oscillating |  |  |  |  |  |  |  |  |
| **Control variables** | |  |  |  |  |  |  |  |
| Network Size | 23.889 | 22.862 | 0 | 167 | 33.454 | 26.377 | 0 | 168 |
| Household Income / 1,000 CLP | 894.70 | 1,062.23 | 20 | 10 | 879.17 | 848.61 | 40 | 10 |
| Female | 0.502 |  | 0 | 1 | 0.504 |  | 0 | 1 |
| Education: Primary | 0.137 |  | 0 | 1 | 0.157 |  | 0 | 1 |
| Education: Secondary | 0.386 |  | 0 | 1 | 0.387 |  | 0 | 1 |
| Education: Vocational | 0.207 |  | 0 | 1 | 0.189 |  | 0 | 1 |
| Education: University | 0.270 |  | 0 | 1 | 0.268 |  | 0 | 1 |

| **Table A1. Continued.** | | | | | | | | |
| --- | --- | --- | --- | --- | --- | --- | --- | --- |
|  | **2021** (*N* = 1,278) | | | | **2023** (*N =* 1,137) | | | |
| **Variable** | Mean | SD | Min | Max | Mean | SD | Min | Max |
| **Class Homogeneity** | |  |  |  |  |  |  |  |
| Class Homogeneity | 0.403 | 0.184 | 0 | 1 | 0.432 | 0.192 | 0 | 1 |
| **Occupational Class** | |  |  |  |  |  |  |  |
| Upper-Middle Class | 0.189 |  | 0 | 1 | 0.191 |  | 0 | 1 |
| Lower-Middle Class | 0.268 |  | 0 | 1 | 0.276 |  | 0 | 1 |
| Working Class | 0.543 |  | 0 | 1 | 0.533 |  | 0 | 1 |
| **Conjunctural Mobility** | |  |  |  |  |  |  |  |
| Upward | 0.176 |  | 0 | 1 | 0.206 |  | 0 | 1 |
| Downward | 0.131 |  | 0 | 1 | 0.137 |  | 0 | 1 |
| Stayers | 0.693 |  | 0 | 1 | 0.657 |  | 0 | 1 |
| **Structural Mobility** | |  |  |  |  |  |  |  |
| Upward | 0.176 |  | 0 | 1 | 0.246 |  | 0 | 1 |
| Downward | 0.131 |  | 0 | 1 | 0.120 |  | 0 | 1 |
| Stayers | 0.693 |  | 0 | 1 | 0.634 |  | 0 | 1 |
| **Cumulative Trajectory** | |  |  |  |  |  |  |  |
| Only Upward |  |  |  |  | 0.109 |  | 0 | 1 |
| Only Downward | |  |  |  | 0.210 |  | 0 | 1 |
| Stable |  |  |  |  | 0.207 |  | 0 | 1 |
| Oscillating |  |  |  |  | 0.473 |  | 0 | 1 |
| **Control variables** | |  |  |  |  |  |  |  |
| Network Size | 36.012 | 25.577 | 0 | 178 | 36.012 | 27.083 | 0 | 189 |
| Household Income / 1,000 CLP | 1,060.26 | 986.74 | 30 | 10 | 1,266.21 | 1,058.95 | 60 | 10 |
| Female | 0.483 |  | 0 | 1 | 0.498 |  | 0 | 1 |
| Education: Primary | 0.093 |  | 0 | 1 | 0.101 |  | 0 | 1 |
| Education: Secondary | 0.379 |  | 0 | 1 | 0.398 |  | 0 | 1 |
| Education: Vocational | 0.198 |  | 0 | 1 | 0.181 |  | 0 | 1 |
| Education: University | 0.330 |  | 0 | 1 | 0.320 |  | 0 | 1 |
| **Note:** ELSOC Survey 2016-2023. Unbalanced panel with 2,520 unique individuals. Panel participation: 26% observed in all 4 waves, 26.3% in 3 waves, 23.3% in 2 waves, 24.4% in 1 wave only. Weighted figures (own computations). For categorical variables, the mean represents the proportion. Conjunctural and Structural Mobility, being variables in reference to 2016, do not have data for that wave. Cumulative Trajectory, being an ex-post construct, only has information from the last wave. | | | | | | | | |

| **Table A2.** Regression models predicting class-based diversity by inter-wave mobility. | | | |  |
| --- | --- | --- | --- | --- |
|  | **Shannon** | **Blau** | **Blau (norm.)** | **HHI** |
| Intercept | 1.318 *** | 0.665 *** | 0.721 *** | 0.335 *** |
|  | (0.029) | (0.010) | (0.011) | (0.010) |
| Class origin: lower-middle (ref. working) | 0.037 | 0.010 | 0.011 | -0.010 |
|  | (0.024) | (0.008) | (0.009) | (0.008) |
| Class origin: upper-middle (ref. working) | 0.085 * | 0.027 * | 0.029 * | -0.027 * |
|  | (0.040) | (0.013) | (0.014) | (0.013) |
| Inter-wave mobility: Upward (re.vs stayer) | 0.099 *** | 0.026 *** | 0.028 *** | -0.026 *** |
|  | (0.015) | (0.005) | (0.006) | (0.005) |
| Inter-wave mobility: Downward (ref.vs stayer) | 0.057 *** | 0.014 * | 0.015 * | -0.014 * |
|  | (0.017) | (0.006) | (0.006) | (0.006) |
| Network size (within) | 0.008 *** | 0.002 *** | 0.002 *** | -0.002 *** |
|  | (0.000) | (0.000) | (0.000) | (0.000) |
| Income (within) | 0.000 | -0.000 | -0.000 | 0.000 |
|  | (0.000) | (0.000) | (0.000) | (0.000) |
| Network size (between) | 0.012 *** | 0.003 *** | 0.003 *** | -0.003 *** |
|  | (0.000) | (0.000) | (0.000) | (0.000) |
| Income (between) | -0.000 | -0.000 | -0.000 | 0.000 |
|  | (0.000) | (0.000) | (0.000) | (0.000) |
| Woman (ref. man) | -0.018 | -0.002 | -0.003 | 0.002 |
|  | (0.020) | (0.007) | (0.007) | (0.007) |
| Education: secondary (ref. primary) | 0.111 *** | 0.019 * | 0.020 * | -0.019 * |
|  | (0.025) | (0.009) | (0.009) | (0.009) |
| Education: vocational (ref. primary) | 0.117 *** | 0.023 * | 0.025 * | -0.023 * |
|  | (0.030) | (0.010) | (0.011) | (0.010) |
| Education: tertiary (ref. primary) | 0.142 *** | 0.027 * | 0.029 * | -0.027 * |
|  | (0.032) | (0.011) | (0.012) | (0.011) |
| Year: 2021 (vs baseline) | 0.049 *** | 0.018 *** | 0.019 *** | -0.018 *** |
|  | (0.011) | (0.004) | (0.004) | (0.004) |
| Year: 2023 (vs baseline) | 0.062 *** | 0.016 *** | 0.018 *** | -0.016 *** |
|  | (0.012) | (0.004) | (0.005) | (0.004) |
| AIC | 7.511.308 | -444.436 | 153.483 | -444.436 |
| BIC | 7.617.142 | -338.602 | 259.317 | -338.602 |
| Log-likelihood | -3.738.654 | 239.218 | -59.742 | 239.218 |
| Num. obs. | 3.735 | 3.735 | 3.735 | 3.735 |
| Num. groups (id) | 1.8743.735 | 1.8743.735 | 1.8743.735 | 1.8743.735 |
| **Note:** ELSOC Survey 2016-2023. Weighted figures. ****p*<0.001, ***p*<0.01, **p*<0.05. | | | |  |

| **Table A3.** Regression models predicting class-based diversity by mobility from class of origin. | | | |  |
| --- | --- | --- | --- | --- |
|  | **Shannon** | **Blau** | **Blau (norm.)** | **HHI** |
| Intercept | 1.328 *** | 0.667 *** | 0.723 *** | 0.333 *** |
|  | (0.029) | (0.010) | (0.011) | (0.010) |
| Class origin: lower-middle (ref. working) | 0.049 | 0.012 | 0.013 | -0.012 |
|  | (0.025) | (0.008) | (0.009) | (0.008) |
| Class origin: upper-middle (ref. working) | 0.096 * | 0.028 * | 0.031 * | -0.028 * |
|  | (0.041) | (0.014) | (0.015) | (0.014) |
| Mobility from origin: Upward (vref.s stayer) | 0.056 *** | 0.015 ** | 0.016 ** | -0.015 ** |
|  | (0.016) | (0.006) | (0.006) | (0.006) |
| Mobility from origin: Downward (refvs stayer) | 0.002 | 0.003 | 0.003 | -0.003 |
|  | (0.022) | (0.008) | (0.008) | (0.008) |
| Network size (within) | 0.008 *** | 0.002 *** | 0.002 *** | -0.002 *** |
|  | (0.000) | (0.000) | (0.000) | (0.000) |
| Income (within) | 0.000 | -0.000 | -0.000 | 0.000 |
|  | (0.000) | (0.000) | (0.000) | (0.000) |
| Network size (between) | 0.012 *** | 0.003 *** | 0.003 *** | -0.003 *** |
|  | (0.000) | (0.000) | (0.000) | (0.000) |
| Income (between) | -0.000 | -0.000 | -0.000 | 0.000 |
|  | (0.000) | (0.000) | (0.000) | (0.000) |
| Woman (ref. man) | -0.018 | -0.002 | -0.002 | 0.002 |
|  | (0.020) | (0.007) | (0.007) | (0.007) |
| Education: secondary (ref. primary) | 0.112 *** | 0.019 * | 0.021 * | -0.019 * |
|  | (0.025) | (0.009) | (0.009) | (0.009) |
| Education: vocational (ref. primary) | 0.114 *** | 0.022 * | 0.024 * | -0.022 * |
|  | (0.030) | (0.010) | (0.011) | (0.010) |
| Education: tertiary (ref. primary) | 0.148 *** | 0.029 ** | 0.031 ** | -0.029 ** |
|  | (0.032) | (0.011) | (0.012) | (0.011) |
| Year: 2021 (vs baseline) | 0.047 *** | 0.017 *** | 0.019 *** | -0.017 *** |
|  | (0.012) | (0.004) | (0.004) | (0.004) |
| Year: 2023 (vs baseline) | 0.059 *** | 0.015 *** | 0.017 *** | -0.015 *** |
|  | (0.012) | (0.004) | (0.005) | (0.004) |
| AIC | 7.544.248 | -426.355 | 171.564 | -426.355 |
| BIC | 7.650.082 | -320.522 | 277.397 | -320.522 |
| Log-likelihood | -3.755.124 | 230.178 | -68.782 | 230.178 |
| Num. obs. | 3.735 | 3.735 | 3.735 | 3.735 |
| Num. groups (id) | 1.8743.735 | 1.8743.735 | 1.8743.735 | 1.8743.735 |
| **Note:** ELSOC Survey 2016-2023. Weighted figures. ****p*<0.001, ***p*<0.01, **p*<0.05. | | | |  |

| **Table A4.** Regression models predicting class-based diversity by cumulative class trajectories. | | | |  |
| --- | --- | --- | --- | --- |
|  | **Shannon** | **Blau** | **Blau (norm.)** | **HHI** |
| Intercept | 1.275 *** | 0.643 *** | 0.696 *** | 0.357 *** |
|  | (0.042) | (0.014) | (0.015) | (0.014) |
| Trajectory: Stable lower-middle class ((ref. Stable working class) | 0.073 | 0.027 | 0.029 | -0.027 |
|  | (0.054) | (0.018) | (0.019) | (0.018) |
| Trajectory: Stable upper-middle class (ref. Stable working class) | 0.037 | 0.022 | 0.024 | -0.022 |
|  | (0.070) | (0.023) | (0.025) | (0.023) |
| Trajectory: Only upward (ref. Stable working class) | 0.104 ** | 0.027 * | 0.029 * | -0.027 * |
|  | (0.037) | (0.012) | (0.013) | (0.012) |
| Trajectory: Only downward (ref. Stable working class) | 0.066 | 0.009 | 0.010 | -0.009 |
|  | (0.043) | (0.014) | (0.015) | (0.014) |
| Trajectory: Oscillating (ref. Stable working class) | 0.184 *** | 0.055 *** | 0.060 *** | -0.055 *** |
|  | (0.036) | (0.012) | (0.013) | (0.012) |
| Network size (between) | 0.011 *** | 0.002 *** | 0.003 *** | -0.002 *** |
|  | (0.001) | (0.000) | (0.000) | (0.000) |
| Income (between) | -0.000 | -0.000 | -0.000 | 0.000 |
|  | (0.000) | (0.000) | (0.000) | (0.000) |
| Woman (ref. man) | -0.056 * | -0.016 * | -0.017 * | 0.016 * |
|  | (0.024) | (0.008) | (0.009) | (0.008) |
| Education: secondary (ref. primary) | 0.288 *** | 0.082 *** | 0.089 *** | -0.082 *** |
|  | (0.042) | (0.014) | (0.015) | (0.014) |
| Education: vocational (ref. primary) | 0.370 *** | 0.101 *** | 0.109 *** | -0.101 *** |
|  | (0.051) | (0.017) | (0.018) | (0.017) |
| Education: tertiary (ref. primary) | 0.381 *** | 0.102 *** | 0.111 *** | -0.102 *** |
|  | (0.051) | (0.017) | (0.018) | (0.017) |
| AIC | 2.835.004 | 372.454 | 551.589 | 372.454 |
| BIC | 2.900.266 | 437.716 | 616.852 | 437.716 |
| Log-likelihood | -1.404.502 | -173.227 | -262.795 | -173.227 |
| Num. obs. | 1.119 | 1.119 | 1.119 | 1.119 |
| Num. groups (id) |  |  |  |  |
| **Note:** ELSOC Survey 2016-2023. Weighted figures. ****p*<0.001, ***p*<0.01, **p*<0.05. | | | |  |

**Figure A1.** Class trajectories across panel waves (Unbalanced panel)

**Note**: ELSOC Survey 2016-2023. Unbalanced panel (all observed individuals per wave). Weighted estimates. Flows end at last observed wave for attrited individuals.
